# Socioeconomic Patterns of Tobacco Use–An Example from the Balkans

**DOI:** 10.3389/fphar.2016.00372

**Published:** 2016-10-19

**Authors:** Dragan Vasiljevic, Natasa Mihailovic, Snezana Radovanovic

**Affiliations:** ^1^Department of Hygiene and Human Ecology, Faculty of Medical Sciences, University of Kragujevac and Institute of Public Health KragujevacKragujevac, Serbia; ^2^Center for Informatics and Biostatistics, Institute of Public Health KragujevacKragujevac, Serbia; ^3^Department of Social Medicine, Faculty of Medical Sciences, University of Kragujevac, Kragujevac and Institute of Public Health KragujevacKragujevac, Serbia

**Keywords:** smoking cigarettes, socioeconomic factors, National Health Survey, Serbia, socioeconomic inequality

## Inequalities in patterns of tobacco use

The number of smokers in the world is about 1.4 billion and projections are that this number is going to reach about 1.8 billion by the year 2030 (Bosdriesz et al., 2014). In many countries, smoking is known to be the single biggest source of inequalities in mortality and morbidity among the rich and the poor (Yamada et al., 2013). Some independent studies at both national and international levels have shown a connection between the use of tobacco and social and economic factors such as nationality, place of living, profession, education, gender, and age. Inequalities in socioeconomic status and its impact on people's health represents a global issue (Guo and Sa, 2015; Radevic et al., 2016). In most high-income countries today, there is a negative gradient in smoking, and smoking is more common among countries of low socio-economic status. As a result, smoking is one of the most important factors contributing to health inequalities (Bosdriesz et al., 2014).

## The data report methods

### Public data set description—Serbian 2013 national health survey

The study of health of population in Serbia conducted in 2013 was the source of used data. This was the third national population health survey conducted by the Ministry of Health of the Republic of Serbia (IPHS, 2013). The first such survey was conducted in 2000 and another one in 2006. In the third Survey, a harmonization of research tools (methodology, questionnaires, instructions) with the instruments of the European Health Survey second wave (EHIS wave 2; EHIS, 2013) was carried out in order to achieve the highest degree of comparability of results with the countries members of the European Union, according to a defined, internationally accepted indicators (ECHI, OMC, WHO, UNGASS, MD).

Health Survey of the Serbian population was carried out through interviews, anthropometric measurements and blood pressure measurements. The target population were people 15 or more years of age who were living in private households on the territory of the Republic of Serbia at the time of data collection. Categories of persons who belonged to the target population, and who were not included in the study population, were persons living in collective households and institutions, as well as persons living on the territory of the Autonomous Province of Kosovo and Metohija, which is under the authority of UNMIK (UN Mission in Kosovo).

The study used the most complete population register that includes a sampling units defined within the target population—Census of Population, Households, and Dwellings in the Republic of Serbia conducted in 2011. In accordance with the recommendations for the implementation of population health research EUROSTAT, the European Health Research—Second Wave—Methodological guide (EHIS wave 2, Methodological manual) the National representative probability sample was used: Two-stage stratified sample with a known probability of selection of sample units at every stage sampling. The sample was drawn so as to provide a statistically reliable estimation of a great number of indicators of population health condition at the national level, considering geographic areas/statistical regions of Belgrade, Vojvodina, Shumadija, Western, Southern, and Eastern Serbia, and at the level of urban and other settlements/areas. The mechanisms that have been used to obtain a random sample of households and respondents represent a combination of the two sampling techniques: Stratification and multi-stage sampling. Population data for Serbia were used in order to make the initial strata. Also, two variables were used in order to create strata and to assess their size and the percentage distribution of the sample—region (four territorial strata according to NUTS2) and type of settlement, or division of settlements into urban and other settlements. The variables: Region and type of settlement were simultaneously used both for stratification of the population, and for stratification of the sample and therefore the samples are stratified in two dimensions. As the main strata in the sample four statistical regions were identified: Vojvodina, Belgrade, Shumadija, and Western Serbia, Southern and Eastern Serbia. The further division of four strata in urban and other areas gave a total of eight strata in Serbia.

Units of the first stage were selected based on probability proportional to their size (Probability Proportional Sampling-PPS). In the first stage, a total of 670 EAs stratum was selected. The units of the second stage were households. Lists of all households in selected EAs stratums were updated before the final selection of households. After completion of the update, 10 households along with three reserve households were selected within each enumeration area. Selected households were chosen using a linear random beginning sampling method with the equal steps of choice (Simple Random Sample Without Replacement-SRSWoR). In this way, households were selected with equal probability of selection without repetition. The sample was selected so as to provide a statistically valid estimation of Serbia as a whole and then at the level of individual regions (Belgrade, Vojvodina, Shumadija, and Western Serbia, Southern and Eastern Serbia), as well as at the level of a single type of settlement (urban, rural). Starting with the precision requests for the assessment and the level of obtaining reliable assessments, and in accordance with the recommendations for the implementation of population health research, the number of respondents who would provide the required sample size by strata was planned. A sample of 6700 households with 19,284 members expected was planned. A sample of 6500 households in which there were 19,079 listed members was realized.

Three types of questionnaires were used in the survey: Questionnaire for Household—collecting information on all household members, the characteristics of the household, as well as on the characteristics of the household residence. The questionnaire had to be completed in the course of verbal communication between the interviewers and interviewees who represented the main person in the household to answer questions of interest. The questionnaire “face to face” is to be filled in with each member of the household. Self-administered questionnaire which should be filled in by each household member aged 15 and over without the participation of the interviewer. This type of questionnaire was used because it was estimated that the questions concerning sensitive items of alcohol use, sexual behavior and so on were not suitable for filling by method “face to face.” In order to complete the questionnaires a method of computer-assisted personal interviewing–CAPI was used as well as the process of interviewing through paper-and-pencil procedures–PAPI for self-administered questionnaire.

Fieldwork was conducted in the period from 7 October to 30 December 2013, which respected the legislation relating to the European Health Research—second cycle: The collection of data in the field should take at least 3 months of which at least 1 month should be in the period from September to December, or in the fall. In order to achieve a high level of quality of the collected data, to provide a high response rate of households and in order to protect the representativeness of the sample, the election and training of interviewers had been organized prior to the commencement of field work, and also guidelines for the monitoring and control of field work were given to them. 68 teams with a total of 204 interviewers were formed to perform the fieldwork. Each team consisted of three members, one of which had to be a health worker or a doctor or a nurse-technician. 13 field supervisors were responsible for monitoring and control of field work. The control procedures of the whole process of research, in all its phases, included the control of sampling and control of work in the field. At the end of the field work phase the supercontrol was performed. In order to carry this out, 10% of EAs were randomly selected from the total sample. Supercontrol results showed that data collection procedures went well, which means that both the interviewers and supervisors followed the instructions received during the training process.

Ethical Standards in Health Research were harmonized with the international World Medical Association Declaration of Helsinki. In order to respect the privacy of the subjects of research and confidentiality of information collected, all necessary steps in accordance with the Law on Personal Data Protection (Off. Gazette of RS No 97/08, 104/09)^1^ were taken. Field researchers were required to give a printed document that informed research participants about the Research (Notice of Survey signed by the Minister of Health) and the approval of the Ethics Committee on its implementation, on the rights of patients, and about where and how they can submit complaint/grievance if estimate that their rights have been in any way compromised. Also, interviewers needed to obtain the signed informative consent of each of the participants for accepting to participate in the survey. In research, the collection of data that identify the respondents was avoided to the greatest possible extent (necessary identifiers were removed at the earliest stage of statistical analysis and replaced with code).

## Survey data description

Out of total of 10,089 households contacted, 6500 of them agreed to participate in the survey, so that the response rate of households was 64.4%. Out of total of 16,474 registered household members aged 15 years and over, 14,623 of them agreed to be interviewed, giving a response rate of 88.9%. Out of this number of people who agreed to be interviewed, 13,756 of them accepted to fill in the questionnaire (response rate 94.1%). For the purposes of this study, the data on households and population age 15 and over were used, so that the final sample for analysis included 6834 patients (aged 20 and over).

Of the independent variables, the researchers used demographic characteristics (age, gender, type of settlement, and marital status) and socioeconomic status (education, employment, and well-being index). Participants' age was categorized in to 8 age groups (20–24, 25–34, 35–44, 45–54, 55–64, 65–74, 75–84, and 85 years or more). Gender is coded as male and female, place of residence as urban and rural, while the marital status was categorized as marriage or common law marriage and not married, divorced or widowed. Variables that reflect the socio-economic situation are education, which is designated as higher, secondary and elementary, employment status as employed and unemployed and household. The Wealth Index is based on household assets and housing characteristics, such as the possession of color TV set, cell phone, refrigerator, dish washer, washing machine, PC, AC, car, construction material of floors, roofs and walls, the number of bedrooms per household member, type of drinking water resources and sanitation facility as well as heating fuel and Internet access. Based on the Wealth Index, households were classified into five groups of equal size–quintiles (1) the poorest (Q1), (2) poorer (Q2), (3) middle (Q3), (4) richer (Q4), and (5) the richest (Q5). For the purposes of this paper, respondents were classified into three socio-economic categories: Poor class, middle class, and rich class. The use of tobacco, as a dependent variable in this analysis, refers to smoking every day and occasionally.

The data set has been submitted in a public repository Figshare and it is available on: https://figshare.com/s/6ca73e2f4911b89b50f7. Data has been uploaded as Excel file while questionnaires are in PDF formats. Readers can retrieve and reuse publicly available information by visiting links given above.

## Socioeconomic factors associated with tobacco use

The highest percentage of respondents belonged to the age group of 55–64 years (22.8%). There were more men (53.9%) than women (46.1%) in the sample. The highest percentage of respondents has completed secondary education (61.2%), while there is the least of those who have high education (16.4%). In relation to the employment status the highest percentage belongs to the group of inactive population (60.3%). More than half of the respondents live in urban areas (59.8%). When it comes to well-being index, the largest percentage of respondents belongs to the middle class (61.3%).

Results of binary logistic regression showed that cigarette smoking is under the influence of age, gender, marital status, education and well-being index (Table 1). The prevalence of cigarette consumption in males is 50.7%, whereas males are 1.3 times more likely to use tobacco than females. Compared to the younger population (< 24 years) members of the group of the elderly population (25 and older) are more likely to consume cigarettes. Married or living with a partner are 1.4 times more likely to smoke compared to not married, divorced, widowed. Respondents with higher education have 1.8 times greater chance of cigarette smoking compared to those with low education (OR = 1.851). For an index of wealth, odds ratios are calculated by taking the poorest category of wealth as a reference. Members of rich class are more often smokers (OR = 1.436) compared with those who belong to a poor class of the population.

**Table 1:** **Binary logistic regression of factors associated with tobacco use in Serbia**.

**Variables**	***N***	**%**	**Binary logistic regression**	***p***
			**OR (95% CI)**	
**GENDER**				
Female	2185	49.3	1	<0.001
Male	2248	50.7	1.298 (1.166–1.444)	
**Age (Years)**			1.036 (1.032–1.040)	<0.001
**MARITAL STATUS**				
Not married, divorced, widowed	1468	40.9	1	
Married or living with a partner	2965	66.9	1.253 (1.115–1.409)	<0.001
**EDUCATION**				
Low	1019	23.0	1	
Middle	2779	62.7	1.329 (1.154–1.531)	<0.001
High	635	14.3	1.851 (1.534–2.233)	<0.001
**EMPLYMENT STATUS**				
Employment	1884	42.5	1	
Unemployment	2549	57.5	1.123 (0.994–1.267)	>0.05
**TYPE OF SETTLEMENT**				
Urban	2622	59.1	1	
Rural	1811	40.9	1.128 (0.096–1.277)	>0.05
**WELL-BEING INDEX**				
Poor class	907	20.5	1	
Middle class	2737	61.7	1.156 (0.992–1.349)	>0.05
Rich class	789	17.8	1.436 (1.168–1.765)	<0.05

## Landscape of tobacco addiction across the globe

Aforementioned dataset showed that there are significant differences in the consumption of cigarettes depending on the demographic and socioeconomic characteristics of the respondents. They are consistent with the findings of other studies that show that the prevalence of cigarette consumption varies among regions of the world, considerably depending on gender, level of education and well-being index (Janković and Simić, 2012). Also, smoking is a behavior that has the greatest impact on health inequalities (Giovino et al., 2012).

In most countries, smoking is more prevalent among men than women as represented in the Global Adult Tobacco Survey conducted in Brazil, Mexico, Philippines, Uruguay, Bangladesh, China, Thailand, India, Turkey, Egypt, Poland, Russia, Ukraine, and Vietnam (Barbeau et al., 2004). Also, the prevalence of tobacco consumption is increasing with age in almost all countries included in the Global Adult Tobacco Survey, except in Mexico and Poland where age group has no influence, while the trend of tobacco consumption decreases with increasing age in the Russian Federation (Jakovljevic and Milovanovic, 2015), Ukraine and Uruguay. The trend shows a significant reduction in the incidence of tobacco use by increasing the level of education in Bangladesh, Egypt, India, the Philippines and Thailand, Poland, the Russian Federation, China, Ukraine, and Vietnam (Palipudi et al., 2012). Other studies also have shown that tobacco consumption was significantly higher among older, poorer and less educated population. Smoking prevalence also varies depending on the individual and sociocultural characteristics (Rani et al., 2003).

The correlation between tobacco use among men and women of Asian countries, with socio-economic and demographic factors, showed that the inhabitants of rural areas were more likely to smoke. Older men are more likely to smoke in most countries. Also, smokers are more likely to be married men and married women. Smoking is strongly associated with education and with the level of wealth in many Asian countries. Individuals who were educated and wealthier individuals were less likely to smoke. Smoking has been associated with religion (Agrawal et al., 2013; Sreeramareddy et al., 2014).

The trend of reducing the chances of tobacco products use with the increase of wealth existed in results obtained in populations of Bangladesh, India, the Philippines, Thailand, Turkey, Ukraine, Uruguay, and Vietnam (Rancic and Jakovljevic, 2016). However, in several countries wealth and higher level of education did not result in decreased use of tobacco, such as Mexico where tobacco use was actually lower among poor people and in China where the lowest rate of tobacco use was present among both the poorest and the wealthiest (Palipudi et al., 2012; Jakovljevic, 2014). Correlation between smoking and socio-economic status among Chinese smokers has shown that the poorest and uneducated or illiterate people smoked 11% and 14% more than those who earned more or who had higher level of education (Guo and Sa, 2015). And other studies that dealt with the connection of smoking with socio-economic status also demonstrate that cigarette smoking is more prevalent among low educated and poorer people (Nédó and Paulik, 2012).

## Conclusive remarks

Social differences in smoking behavior could make the existing social inequalities related to health even worse. Therefore, policies and interventions which promote cessation of smoking should pay more attention to the disadvantages social groups. Policymakers should consider the socio-economic importance of tobacco use in the design, implementation and evaluation of tobacco control interventions.

## Author contributions

All authors listed, have made substantial, direct and intellectual contribution to the work, and approved it for publication. DV and SR developed research questions, designed the study, and prepared manuscript for this Data report. NM contributed through data analysis and interpretation.

### Conflict of interest statement

The authors declare that the research was conducted in the absence of any commercial or financial relationships that could be construed as a potential conflict of interest.

## Footnotes

^1^http://www.refworld.org/pdfid/4b5718f52.pdf
